# Penile Prosthesis: What Should We Do about Complications?

**DOI:** 10.1155/2008/573560

**Published:** 2008-11-04

**Authors:** C. Bettocchi, P. Ditonno, F. Palumbo, G. Lucarelli, G. Garaffa, B. Giammusso, M. Battaglia

**Affiliations:** ^1^Department of Emergency and Organ Transplantation, Urology Andrology and Kidney Transplantation Unit, University of Bari, 70124 Bari, Italy; ^2^St Peter's Department of Andrology, University College London Hospitals, London W1G 6BJ, UK; ^3^Department of Urology, Vittorio Emanuele Hospital, University of Catania, 95124 Catania, Italy

## Abstract

Even in the era of phoshodiesterase type 5 inhibitors, penile implants are considered the definitive solution for the treatment of organic erectile disfunction. The advent of new surgical tools and new infection-resistant materials has significantly reduced the risk of intra and post-operative complications and the need for revision surgery. Various companies have also improved their mechanical systems in order to reduce the risk of failures, and their products are now so good they may last lifelong. In this article, we evaluate the intraoperative and postoperative complications recorded in our experience and in literature reports, and make some suggestions as to how to prevent or correct them.

## 1. INTRODUCTION

Nowadays implanting a penile prosthesis is the definitive solution for the treatment of organic erectile dysfunction (ED), even in the era of effective and safe oral medications [1]. The types of prosthesis most commonly implanted are the three-piece inflatable device, the two-piece inflatable device, and the soft and malleable prosthesis. In the last few years, the three-piece inflatable device has been used for preference, as it improves the erection, the flaccid, and appearance of the penis and as it yields a more acceptable and cosmetical functional results [2]. On the other hand, the relative complexity of this last device is also the source of mechanical failures and patients' difficulties in managing the device. In the last decade, there has been a continuous improvement in the mechanical function of the devices and in the composition of the materials used but device-related complications still occur.

Some complications can be prevented by a correct preoperative assessment. The surgeon has to understand the patient's real needs and expectations, as well as those of his partner in order to be able to choose the right device. The counselling must also include a complete, clear explanation of how the device functions and the obvious changes that will arise in the sexual life of the couple. Informed consent to the procedure is mandatory, and when discussing the option of a penile implant with the patient, issues such as complications and the irreversibility of the procedure should be exhaustively discussed.

In this paper, we evaluate the intraoperative and postoperative complications recorded in our experience and in literature reports, and make some suggestions as to how to prevent or correct them.

## 2. INTRAOPERATIVE COMPLICATIONS

### 2.1. Cylinders positioning

During the implant procedure, after having exposed the corpora cavernosa and performed the corporotomy, the first critical step is dilating the corpora. In most patients the corpus cavernosum cavity is dilated to the maximum capacity using Hegar dilators of various sizes. The dilator must be introduced through the corpus by pushing it in an outward direction in order to avoid cross-over perforation. In cases of fibrotic corpora, special dilators may be useful to create an appropriate space (Rossello dilators or Otis urethrotome) because perforation is especially risky in this case.

A distal corpora perforation can be corrected first of all by exposing the damaged corpus apex. Then, if it is only a small hole, the tip can be closed with separate PDS stitches. The way to manage distal perforation in cases of larger holes is by covering the damaged apex with a dacron or gore-tex sleeve.

**Figure 1:**
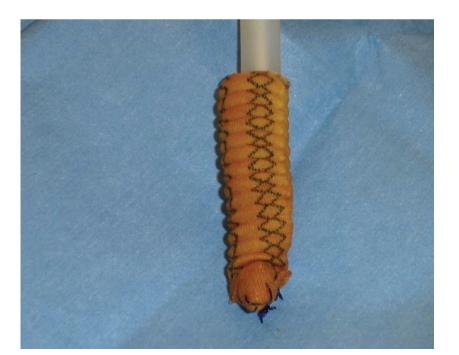
A dacron sock created around the tip of a malleable prosthesis in a case of proximal corpus cavernosum perforation.

Proximal corpora perforation usually occurs during dilatation of the corpus cavernosum crura. A possible way to evaluate a proximal perforation intraoperatively is by positioning dilators in both crura and checking whether they are at different heights, showing that one has penetrated too deeply inside the corpus. If not discovered during the operation, a postoperative MRI scan is the best evaluation to confirm a proximal perforation. One of the two ways of managing this complication is by creating dacron or gore-tex socks, especially in cases of a malleable or soft prosthesis (Figure 1). The other possibility, indicated for inflatable devices, is to fix the cylinders to the surrounding corpora tissue, placing stitches above and below the tubes input. The anchored cylinder tends not to protrude, allowing healing of the perforation. Another similar solution involves fashioning a sling through the tip extender using nonabsorbable sutures.

Incorrect introduction of the dilators is the main cause of cross-over perforation. It is important to recognise this kind of perforation as soon as possible so as to implant two cylinders in the same corpus. Usually a redo correct ipsilateral dilatation is sufficient to correct the cross-over perforation.

Another consequence of incorrect dilators introduction is urethral perforation. To check for urethral injuries, it is always best to irrigate the corpora with a saline plus antibiotic solution: if the fluid leaks through the urethral meatus, a perforation has occurred. The diagnosis can be confirmed by cystoscopy. The treatment option in such cases is urethral repair for proximal perforations. If the laceration involves the urethral meatus, it is advisable to postpone the procedure. It is possible to position a urethral catheter if necessary with a suprapubic catheter, delaying insertion of the cylinder or positioning of a malleable prosthesis until the damaged urethra has healed. The malleable prosthesis will be replaced by the inflatable cylinder at a later date during a second operation.

A rare complication has been described by Hatzimouratidis et al. [3]; it occurred during dilation of the corpora cavernosa with Brooks dilators: the head detached and stuck to the tip of the corpus cavernosum. The case was managed by incising the distal lateral part of the corpora cavernosa and then removing the head of the dilators. In any case, we strongly recommend examining all surgical tools carefully before using them.

### 2.2. Reservoir positioning

The possible complications occurring during the reservoir positioning step are mostly due to this peculiar blind procedure. If the fascia is not completely opened, the reservoir may not pass through, remaining outside: this is a typical postoperative complication. Another possibility is to open the peritoneum: in this case, it is mandatory to check for bowel injuries.

During reservoir positioning, it is very important to have positioned a urethral catheter and ensured that the patient has completely emptied his bladder. If not, the risk of bladder perforation is high. This complication can also occur in patients who have previously undergone pelvic surgery, such as radical prostatectomy. If a bladder perforation occurs, cystoscopy can confirm the damage severity; usually leaving a catheter in place for a few days is sufficient to treat such complications. In rare cases of wide perforation, an open bladder repair can be performed.

### 2.3. Component failure/breakage

In order to avoid a malfunctioning device, it is always advisable to check correct device functioning before placement and to activate the pump with cylinders connected after the placement. At this surgical stage, it is easy to substitute a nonfunctioning device.

Another possible complication is breakage of device components during cavernotomy closure or during repositioning of Scott retractor's hooks during the operation. One way to prevent device perforation is to put the stitches in before performing the corporotomy and before positioning the cylinders.

## 3. POSTOPERATIVE COMPLICATIONS

### 3.1. Cylinders complications

**Infections**

Infection is one of the most fearsome complications, having an incidence of 8 to 20%, as reported in large series of implants [2–4]. Infections can occur a few months after surgery and a typical sign is persistent, unchanging, or even increasing pain. The pain could be exacerbated by activating the device. Other signs of infection are penile or scrotal erythema, fever, purulent drainage from the wound, or skin erosion. Diabetic patients are more likely to develop an infection, even if the previous concept that poor glycemic control increases the risk has not been confirmed [5]. Moreover, insulin dependency and hemoglobin A1C serum levels are not considered additional risk factors. Other conditions, possibly associated with an increased risk of infection, are the use of immunosuppressive drugs and steroids, and the presence of spinal cord injury.

When the presence of infection is confirmed, the use of systemic antibiotics therapy is not sufficient in the vast majority of cases. This is due to the infectious agent's ability to create a biofilm surrounding the prosthesis components, protecting bacteria from the antibiotic action. In most cases, the infection is sustained by opportunistic bacteria such as *Staphylococcus epidermidis* or *Streptococcus agalactie*; more rarely, toxic bacteria like *Escherichia coli*, *Staphylococcus aureus*, *Enterococcus faecalis*, or Pseudomonas are involved. The latter agents tend to present early in the postoperative period, with fever, deep tissue penetration, and abundant purulent drainage.

The classical approach to an infected device is the immediate removal of all the components and placement of a new implant after some delay for healing. The advantage of this solution is that the new implant is scheduled only when the infection has completely cleared. The main disadvantage is the scarring process that occurs inside the penis and hence penile retraction causing more difficult surgery later. In the last years salvage procedures have been proposed that allow positioning of a new penile prosthesis at the same time as removal of the infected one [6, 7]. The immediate salvage procedure consists of removal of the infected prosthesis and wound irrigation with seven different antiseptic solutions including antibiotics (Kanamycin, Bacitracin, Vancomycin, and Gentamycin), hydrogen peroxide, and betadine. A new prosthesis is then easily placed, and the overall success rate is more than 80%. The delayed salvage procedure consists of placement of a drainage tube after removal of the prosthesis; antibiotic solution is irrigated through the drain and a new prosthesis is placed about 3 days later. Actually, no advantage has been demonstrated for the delayed salvage procedure over the immediate one. A few years ago, based on the evidence that some antibiotics are particularly indicated to protect silicone graft materials, the American Medical System Company developed a minocycline-rifampicin-coated penile prosthesis called Inhibizone [8]. Early experiences with this new device have demonstrated an evident reduction of overall infections, and no infections at all in primary implanted patients [9]. Another local approach to prevent device infection has been proposed by the Mentor Corporation Company and consists of applying a special hydrophilic coating that seems to inhibit bacterial adherence. The prosthesis is then soaked in antibiotics and the combined effect should reduce the risk of infection. In an initial experience, the Mentor Titan prosthesis has also demonstrated effectiveness in reducing the infection rate [10].

**Figure 2:**
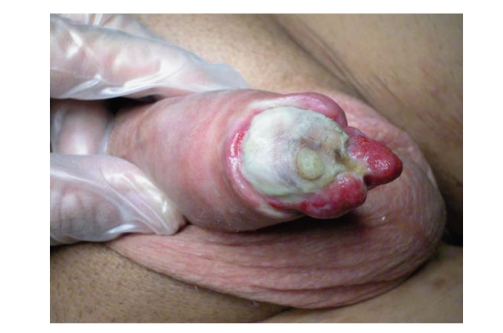
Distal erosion with massive glans necrosis.

**Figure 3:**
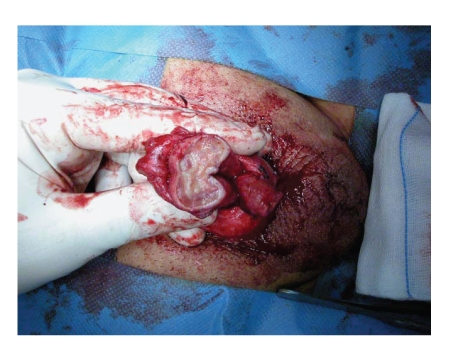
Penile amputation to eliminate all the necrotic tissue surrounding the extruded cylinders.

In some patients, the infection could be associated with important tissue necrosis: in this case, a salvage procedure is not advisable. Severe distal tissue necrosis is a dramatic event that may even require penile glansectomy or amputation (Figures 2 and 3) after prosthesis removal.

**Wrong sizing**

**Figure 4:**
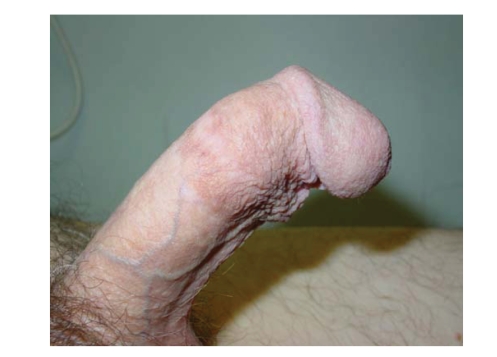
A “Concorde” effect due to undersized cylinders.

Using an oversized cylinder can lead to an S-shaped deformity and buckling. As reported by Moncada et al. [11], an oversized cylinder is responsible for constant pain and exposes the patient to the risk of erosion. The solution in such cases is to replace the device. The opposite problem is undersizing, which will have the effect of a so-called “concorde deformity” (Figure 4) with excess mobility of the glans. In this case, cylinder removal is not necessary and it is possible to mobilize the glans with a subcoronal incision. When the cylinder tip becomes visible, nonabsorbable sutures can be used to hitch the glans and anchor it to the tunica albuginea, in order to completely cover the head of the prosthesis.

**Erosion**

In the era of hydraulic inflatable devices, erosions are considered a rare complication. Distal erosion can be due to an excessive intraoperative corpora cavernosa dilatation, when oversized cylinders are used, in patients with loss of penile sensation (cold glans syndrome) and in patients unable to deflate the device when not in use. To manage distal erosion, it is necessary to remove the cylinder if oversized and replace it with a smaller prosthesis. The new device has to be placed far from the scar tissue, performing a new dilatation. Cavernosa reconstruction can be performed with albugineal surgery, as proposed by Mulcahy [12]. The cylinder can usually be readily reseated in an area of spongy tissue behind the back wall of the sheath containing the extruded cylinder. This is done by making a corporotomy over the cylinder laterally, about half the distance towards the penoscrotal junction, retracting the cylinder to the side, incising the back wall of the cylinder sheath, and dilating a new cavity behind this back wall up to the subglandular area. The cylinder can then be reseated in this new cavity and the back wall of the cylinder sheath will act as the outer covering of the cylinder. A second layer consisting of the outer wall of the cylinder sheath can also be closed to create a more secure barrier against the extrusion of parts. The corporotomy is closed with long-term adsorbable suture. The cylinder is now secured in its proper location by two tough layers comprising the back wall of the original sheath and the corporotomy closure. Cavernosa reconstruction can also be made using synthetic materials like dacron or Gore-Tex.

A peculiar kind of distal erosion is urethral erosion. A possible solution is to remove the cylinder and to position a suprapubic catheter to allow healing of the urethral perforation. A single-stage procedure has been described by Shaeer [13]: having mobilized the glans off the tip of the corpus cavernosum, the caverno-urethral fistula is disconnected and sealed by primary sutures. The perforation on the corpus cavernosum side is corrected by double breasting or by grafting. The prosthesis is then reimplanted.

Proximal erosion and cross-over erosion are usually intraoperative complications. MRI will confirm the diagnosis: the management consists of removal of the protruded cylinder. A cavernosa reconstruction with a dacron sock is necessary before inserting a new prosthesis.

**Mechanical failure**

Cylinders mechanical failure would involve loss of fluid due to breakage, bulging, or aneurysmatic dilatation. The only solution to manage such cases is to remove the broken device and replace it with a new penile prosthesis. The introduction of new covering materials like Parylene has dramatically reduced the risk of cylinders bulging.

### 3.2. Pump complications

Pump infections require the same management as described above for cylinders. Prevention of hematoma and swelling with closed-suction drains has been shown not to increase the infection rate and to promote an earlier recovery time. In a large series of 425 consecutive primary three-piece penile prosthesis implantations, there were a total of 14 (3.3%) infections and three hematomas (0.7%) during a mean follow-up of 18 months [14].

Pump or connecting tubes erosion is usually associated with infections. If the infection is not extensive and not associated with severe tissue necrosis, a salvage procedure can be performed locally and a new pump can be inserted. In cases of considerable loss of tissue, poor patient conditions, and fever, it is advisable to remove the prosthesis and delay the reimplant.

Pump migration or incorrect positioning is mainly due to insufficient closure of the scrotal space. If the pump is no longer useful because of its incorrect position, a new operation is required to fix it in the correct scrotal place.

### 3.3. Reservoir complications

Reservoir complications are not frequent but include positioning of the reservoir over the fascia. Migration is a rare event and usually occurs when a too big space is created through the fascia to access the Retzius space. With a suprapubic incision, the reservoir can be replaced in the correct paravesical space.

A difficult or failed device deflation can be due to pseudocapsule formation around a partially emptied reservoir. To prevent capsule formation, it is usually sufficient to leave the reservoir half-filled for 24 hours after the operation. Early hospital testing of the prosthesis function is also advisable. When a pseudocapsule is present, surgical revision will be needed to access the Retzius space once more, to break the capsule, and to replace the reservoir. If the previous side is no longer available, it is best to replace the reservoir in the other paravesical space or, if necessary, in the peritoneum.

## 4. CONCLUSIONS

Penile prosthesis implantation is a fascinating surgical technique that has gained an important role in the treatment of severe erectile dysfunction. The advent of new surgical tools and new infection-resistant materials has significantly reduced the risk of intra- and postoperative complications and the need for revision surgery. Various companies have also improved their mechanical systems in order to reduce the risk of failures, and their products are now very good as they may last lifelong. Nevertheless, surgical skill and a meticulous respect for sterility rules remain fundamental requirements to guarantee the success of a penile prosthesis implant.
